# Supplementary material for: Attrition in a 30-year follow-up of a perinatal birth risk cohort: factors change with age
Source: PeerJ. 2014 Jul 8;2:e480. doi: 10.7717/peerj.480 (PMC4103077; doi:10.7717/peerj.480)
Supplement: Supplemental Information S1 [file peerj-02-480-s001.pdf]

Background information of the family, child's health adaptation, and school achievement at 9 years of age

Number of siblings
With whom does the child live (both parents, father, mother, someone else)
Are there other adults living in the family (e.g. grandparents)
Number of rooms, kitchen / kitchenette, WC, bathroom(s), sauna
How many persons live in the household?
Father's occupation?
Is the father working: full time / in shifts / at night exclusively / unemployed / pensioned
Fathers education: compulsory only / occupational / high school / academic
Is father in his desired job / occupation? If not, why?
Mother's occupation?
Is the mother working: full time / in shifts / at night exclusively / unemployed / pensioned
Mothers education: compulsory only / occupational / high school / academic
Is mother in her desired job / occupation? If not, why?
What are the arrangements for after-school day care?
Are there any domestic difficulties e.g. serious illness, divorce, drinking problems, frequent moving from one apartment to another?
Hereditary factors among near relatives:

- Left-handed relatives
- Delayed speech
- Delayed walking
- Motor clumsiness
- Poor dexterity
- Dyslexia
- Difficulties in school (hyperactivity etc.)
- Inattention
- Hypacusis or deafness
- Fits, faints, epilepsy
- Psychoses
- Mental handicap
- Color blindness

Child's health

Infections (mumps, varicella, rubella, measles, paratyphoid fever, recurrent respiratory and/or ear infections)
Accidents (what, when, where treated)

- Seizures, fits or faints
- Other diseases e.g. allergies, headaches, stomach aches
- Enuresis
- Testicular retention
- Eyeglasses
- Color blindness

Hospital treatments including surgery
Last doctor's visit and the reason for it
Any current medical (including physical) therapies

School

What is the name of your child's school?
On what class is the child currently?
How many children are on that class?
Does your child like to go to school?
How is your child's school performance in comparison to siblings' and parents' school performance?
Has the child had difficulties with learning or school adaptation?
If your child does not like going to school, what do you think is the reason?
Does your child do homework without reminding or assistance?
What sort of assistance is needed?
How frequently do you have contact with the teacher?
Does your child have several friends? Are they schoolmates / same age / younger / older
Abilities
Does your child independently put clothes on?
Does your child know how to tell the time?
Is your child in your opinion unusually:

- Clumsy
- Nimble
- Handy
- Gauche
- Lively
- Quiet
- Irritable
- Nice

Does your child get distracted from surrounding sounds to an amount that interferes with homework?
Does your child seem to be tired frequently?
Does your child have difficulties in falling asleep?
Does your child frequently ask "What?"?
Does your child forget instructions easily?
Is your child easily nervous about new things?
Does your child get depressed due to minor failure?
Does your child frequently loose interest on chores?
Is your child more difficult to raise up that his/her siblings?

Parents' views of child's schooling and career choice after completing compulsory schooling

Preferred next stage of education?
Has the child already started the next stage ?
Do you think that your child will be able achieve the level of education / occupation he / she is currently planning?
What is the level of education / occupation you think your child is able to achieve?

What are the child’s current educational / occupational activities?

Questions concerning visits and examinations at 5 years of age (4 years previous to this study visit)

What tests or rehabilitation suggestions were given on the basis of the 5-year examination?
Which tests were performed and/or what kind of rehabilitation was given?
Was the child recommended to day-care?
Do you consider the tests and/or rehabilitatipon beneficial for the child?
Did you get sufficient information about the results of the tests?
Has participation to this study affected your attitude to your child e.g. in comparison to siblings?
How did you inform your child about this study visit?

The text above is a translation of the survey form. The original (copyright Katarina Michelsson) is in Finnish language and the translation was done by the first author of the manuscript. This is not an exact translation, it has not been validated, and it is not meant to be used as a survey form.
