# Supplementary material for: Attrition in a 30-year follow-up of a perinatal birth risk cohort: factors change with age
Source: PeerJ. 2014 Jul 8;2:e480. doi: 10.7717/peerj.480 (PMC4103077; doi:10.7717/peerj.480)
Supplement: Supplemental Information S2 [file peerj-02-480-s002.pdf]

## Teacher's questionnaire at 9 years

Assess the child's performance (good / fair / neutral / acceptable / poor) in

- Reading
- Writing
- Arithmetics
- Crafts
- Sport
- Visual arts

Does the child currently get remedial tutoring? If so, what?
Does the child currently get special tutoring for dyslexia?
Does the child currently get special tutoring for speech?
In your opinion, would the child require special tutoring for dyslexia or speech?
Has the child been frequently absent from school / classes / teaching? If so, why?
Special observations e.g. special skills, hyperactivity, easy to cry etc.
How does the child concentrate in class?
Is the child's behavior different from other children's behavior on the same class? If so, in what way?
How is the child accepted by classmates e.g. popular, being bullied etc.?

Which one of the adjectives in the following list of adjective pairs describes the child better? Evaluation is given on a 5-category Likert-scale: Accurately / Fair / Neither / Fair / Accurately

| | |
|---|---|
| open | introverted |
| Persevering, persistent | impatient |
| Slow | Fast |
| Independent | Dependent |
| Irritable | Cheerful |
| Clumsy | Nimble |
| At ease | Restless |
| Attentive | Daydreaming |
| Cheerful | Sad |
| Insecure | Determined |
| Reliable | Unreliable |
| Sociable | Unsociable |
| Changeful | Balanced |
| Enthusiastic | Bored |
| Dexterous | Inept |
| Confident | Submissive |
| Disturbing | compliant |
| Careful | Careless |
| Extroverted | Timid |
| Compliant | Bossy |
| Trustful | Reserved |
| Mannerly | Self-centered |
| Bold | Polite |
| Subservient | Attacking |

Concentration (good vs. poor, evaluation as above)

*The text above is a translation of the survey form. The original (copyright Katarina Michelsson) is in Finnish language and the translation was done by the first author of the manuscript. This is not an exact translation, it has not been validated, and it is not meant to be used as a survey form.*
