# Supplementary material for: Attrition in a 30-year follow-up of a perinatal birth risk cohort: factors change with age
Source: PeerJ. 2014 Jul 8;2:e480. doi: 10.7717/peerj.480 (PMC4103077; doi:10.7717/peerj.480)
Supplement: Supplemental Information S3 — Participation of subjects in the 30-year longitudinal follow-up study of children in risk of neurodevelopmental disorders. Cases excluded due to death or severe handicap are not shown. Total number of potential participants was 994 at 5 and 9 years, and 864 at 16 and 30 years. Attrition thus was 16% at five, 24% at nine, 35% at 16 and 46% at 30 years. Numbers on the left column indicate participation at different age levels. [file peerj-02-480-s003.pdf]

Background information of the family, child’s health adaptation, and school achievement at 16 years of age

Number of siblings

With whom does the child live (both parents, father, mother, someone else)

Are there other adults living in the family (e.g. grandparents)

Number of rooms (kitchen and bathrooms excluded)?

How many persons live in the household?

Father’s occupation?

Is the father working: full time / in shifts / at night exclusively / unemployed / pensioned

Fathers education: compulsory only / occupational / high school / academic

Is father in his desired job / occupation? If not, why?

Mother’s occupation?

Is the mother working: full time / in shifts / at night exclusively / unemployed / pensioned

Mothers education: compulsory only / occupational / high school / academic

Is mother in her desired job / occupation? If not, why?

Are there any domestic difficulties e.g. serious illness, divorce, drinking problems, frequent moving from one apartment to another?

Child’s health

Accidents (what, when, where treated)

Seizures, fits or faints

Other diseases e.g. allergies, headaches, stomach aches

Hospital treatments including surgery

Last doctor’s visit and the reason for it

Any current medical (including physical) therapies

Parents' views of child's schooling and career choice after completing compulsory schooling

Preferred next stage of education?

Has the child already started the next stage ?

Do you think that your child will be able achieve the level of education / occupation he / she is currently planning?

What is the level of education / occupation you think your child is able to achieve?

What are the child's current educational / occupational activities?

The text above is a translation of the survey form. The original (copyright Katarina Michelsson) is in Finnish language and the translation was done by the first author of the manuscript. This is not an exact translation, it has not been validated, and it is not meant to be used as a survey form.

.
