# Supplementary material for: Attrition in a 30-year follow-up of a perinatal birth risk cohort: factors change with age
Source: PeerJ. 2014 Jul 8;2:e480. doi: 10.7717/peerj.480 (PMC4103077; doi:10.7717/peerj.480)
Supplement: Supplemental Information S4 [file peerj-02-480-s004.pdf]

Questionnaire to parents at 16 years

Name, age etc.

Which one of the adjectives in the following list of adjective pairs describes the child better? Evaluation is given on a 5-category Likert-scale: Accurately / Fair / Neither / Fair / Accurately

open	introverted
Persevering, persistent	impatient
Slow	Fast
Independent	Dependent
Irritable	Cheerful
Clumsy	Nimble
At ease	Restless
Attentive	Daydreaming
Cheerful	Sad
Insecure	Determined
Reliable	Unreliable
Sociable	Unsociable
Changeful	Balanced
Enthusiastic	Bored
Dexterous	Inept
Confident	Submissive
Disturbing	compliant
Careful	Careless
Extroverted	Timid
Compliant	Bossy
Trustful	Reserved
Mannerly	Self-centered
Bold	Polite
Subservient	Attacking
Independent	Seeks attention
Well-behaved	Disobedient
Self-controlled	Confrontational
Able to complete chores	Leaves chores unfinished
Well liked	Bullied
Concentrates well	Easily distracted

The text above is a translation of the survey form. The original (copyright Katarina Michelsson) is in Finnish language and the translation was done by the first author of the manuscript. This is not an exact translation, it has not been validated, and it is not meant to be used as a survey form.
