# Supplementary material for: Attrition in a 30-year follow-up of a perinatal birth risk cohort: factors change with age
Source: PeerJ. 2014 Jul 8;2:e480. doi: 10.7717/peerj.480 (PMC4103077; doi:10.7717/peerj.480)
Supplement: Supplemental Information S5 — English translation of the questionnaire form used at 30 years. [file peerj-02-480-s005.pdf]

The questionnaire used at 30 years in the 30 year longitudinal follow-up of the Helsinki Kätilöopisto 1971-1794 birth cohort of children with neurodevelopmental risks.

The questionnaire has been created in Finnish language partly on the ideas of Barkley and Murphy's "A clinical workbook of attention deficit hyperactivity disorder" (Barkley, R. A., & Murphy, K. R. (1998). Attention-Deficit Hyperactivity Disorder: A Clinical 499 Workbook (2nd. ed). New York: The Guilford Press.)

The text below is a translation of the survey form. The original (copyright Katarina Michelsson) is in Finnish language and the translation was done by the first author of the manuscript. This is not an exact translation, it has not been validated, and it is not meant to be used as a survey form.

Background

Marital status:

1. Single
2. Significant other
3. Maried
4. Divorced
5. Widower

Number of children (year of birth)

Housing

1. Parents' home
2. Alone
3. With a friend
4. Significant other
5. Spouse
6. Other

Schooling:

Did you get remedial tutoring or attended a special class?

School grade (average of grades from 9th year diploma)

Diplomas and/or degrees

1. Compulsory 9 years only
2. Matriculation examination after 12 years
3. Occupational
4. Occupational (extended program)
5. Applied university
6. Applied university (extended program)
7. University (BA)
8. University (MA)
9. University (licentiate)
10. PhD

Field of trade
1. Natural resources and science
2. Technical or transport
3. Trade and administration
4. Travel, food and economy
5. Social and health services
6. Education, leisure
7. Security, military

After compulsory schooling, have you started on any higher education program, which you have not completed? y/n

Do you have presently problems with learning?
1. Learning disorder
2. Reading disorder
3. Writing disorder
4. Speech disorder
5. Difficulties with calculus
6. Motor clumsiness
7. Perception problems

Working history

Current work status?
1. Full time
2. Half time
3. Work by hour
4. Unemployed
5. Student
6. Sick leave
7. Disability pension
8. Other

What is you occupation?

How long have you been in your current job?
1. < 3 months
2. 3-12 months
3. 1-3 years
4. More than 3 years

How many jobs have you had altogether (summer jobs included?)
1. 1-3
2. 4-6
3. >6
4. >10

The longest time in the same job (months)?

Have you ever been dismissed from a job due to improper conduct? y/n

(males) Have you completed the compulsory military service? y/n
1. Complete service time
2. I had to discontinue the service
3. I was discharged because of mental reasons
4. I was discharged because of physical reasons
5. I completed civil alternative (nonmilitary) service
6. Conscientious objector

Health and disease

Current health

1. Very good
2. Rather good
3. Fair
4. Bad
5. Very bad

How many times have you visited a doctor during the last year?
1. None
2. 1-3
3. 4-6
4. 7 or more

Current (regular) medication
1. Somatic
2. Antidepressant
3. Sedative
4. Hypnotic
5. Stimulants (methylphenidate; Ritalin)
6. Other

Use of alcohol
1. Never
2. Now and then
3. About once a month
4. Weekly
5. Several times a week
6. Daily

Do you think that you consume too much alcohol? y/n

Do your near ones think that you use too much alcohol? y/n

Do you smoke? If yes, at what age did you start?
1. Never
2. Less than 5 cigarettes a day
3. 5-20 cigarettes a day

4. More than 20 cigarettes a day

What disorders or health problems do you have currently?
1. Headaches / migraines
2. Stomach problems (dyspepsia)
3. Depression
4. Bipolar mood disorder
5. Schizophrenia
6. Obsessions
7. Panic attacks
8. Anxiety
9. Sleep difficulties / disorder
10. Suicidal ideas

Do you use illicit substances?
1. Never
2. Now and the
3. Weekly
4. Daily

What substance?
1. Cannabis
2. Heroin
3. Amphetamine
4. Cocaine
5. LSD
6. Buprenorphine

Social history

How is your mood mostly y/n
1. Good / happy
2. Anxious / nervous
3. My mood keeps changing constantly
4. Sad / depressed
5. Angry / irritated
6. Empty / without feelings

Satisfaction with current life situation
1. Very satisfied
2. Satisfied
3. Partly satisfied, partly dissatisfied
4. Dissatisfied
5. Extremely dissatisfied

Satisfaction with current personal relationships
6. Very satisfied
7. Satisfied
8. Partly satisfied, partly dissatisfied
9. Dissatisfied

10. Extremely dissatisfied

Satisfaction with social support you get currently

11. Very satisfied
12. Satisfied
13. Partly satisfied, partly dissatisfied
14. Dissatisfied
15. Extremely dissatisfied

Is it difficult to make new friends? y/n

Is it difficult to maintain friendship / keep your friends? y/n

Is it hard to control yourself? y/n

Do you have a driver’s license? y/n

In case you do not currently have a driving license, have you ever applied for it? y/n

Have you been fined because of speeding? y/n

Have you driven under the influence of alcohol or illegal substances? y/n

Have you been imprisoned? y/n

If so, at what age?

Trauma

Have you ever had a bone fracture(s) that required treatment given by a doctor? y/n

1. What part(s) of the body
2. Have you ever had a burn that required treatment given by a doctor y/n
3. At what age and how?
4. Was surgical treatment necessary

Have you ever been involved in a traffic accident? y/n

1. I was the driver of a vehicle
2. I was a passenger in a vehicle
3. On a motorcycle
4. On a bicycle
5. As pedestrian
6. Other

If so, did you get injuries that required treatment given by a doctor? y/n

Have you ever had a concussion that required treatment given by a doctor? y/n

Have you ever lost consciousness because of a concussion? y/n

Have you ever had a blunt force trauma to your body? That required treatment given by a doctor y/n

Have you ever had a sports accident that required treatment given by a doctor?

ADHD related questions

Items 56 - 57 of the 30-year questionnaire are the Barkley and Murphy ADHD current symptoms scale – self-report form (Barkley, R. A., & Murphy, K. R. (1998). Attention-Deficit Hyperactivity Disorder: A Clinical 499 Workbook (2nd. ed). New York: The Guilford Press.)
