# Supplementary material for: Attrition in a 30-year follow-up of a perinatal birth risk cohort: factors change with age
Source: PeerJ. 2014 Jul 8;2:e480. doi: 10.7717/peerj.480 (PMC4103077; doi:10.7717/peerj.480)
Supplement: Supplemental Information S6 — Questionnaire of subjects’ plans for future at 16 years. [file peerj-02-480-s006.pdf]

## Questionnaire of education and career plans at 16 years of age

Are you still at school?

If you are still at school, on what grade?

Do you attend a special class (if so, what)?

What was the average of your grades in your last report

What are your plans after completing compulsory education?

What type of occupation would you like to choose? Specify, if possible.

Do you think that you will qualify for the education you desire? If not, please specify why.

What secondary plans dou you have if you do not qualify?

Do you have a job at the moment? (including summer jobs)

What kind of a job is that?

*The text above is a translation of the survey form. The original (copyright Katarina Michelsson) is in Finnish language and the translation was done by the first author of the manuscript. This is not an exact translation, it has not been validated, and it is not meant to be used as a survey form.*
