# Supplementary material for: Attrition in a 30-year follow-up of a perinatal birth risk cohort: factors change with age
Source: PeerJ. 2014 Jul 8;2:e480. doi: 10.7717/peerj.480 (PMC4103077; doi:10.7717/peerj.480)
Supplement: Supplemental Information S7 [file peerj-02-480-s007.pdf]

Variables, which were tested for statistically significant group differences for response or nonresponse at birth (postnatal) and the ages of 5 years, 9 years, 16 years, and 30 years.

	Variable	Type	number of categories
Birth	Distance to hospital	ordinal	3
	Father's social class	ordinal	5
	Marital status	ordinal	3
	Mother's age	continuous	
	Gender	categorical	2
	Birth weight	continuous	
	Weeks of gestation	continuous	
	Small for date	categorical	2
	Large for date	categorical	2
	Apgar score 1 min	continuous	
	Apgar score 5 min	continuous	
	Apgar score 15 min	continuous	
	Weight gain of mother	ordinal	2
	X-ray investigation during pregnancy	categorical	2
	Smoking during pregnancy	categorical	2
	Number of prior miscarriages	continuous	
	Pregnancy complications (composite score)	continuous	
	Toxemia	categorical	2
	Resuscitation postnatally	continuous	
	Hyperbilirubinemia	continuous	
	Respiratory difficulties	continuous	
	Neurological findings	continuous	
	Amniotic fluid (composite score)	continuous	
	Placental abnormalities (composite score)	continuous	
	Cord complications (composite score)	continuous	
	Number of medical procedures (composite score)	continuous	
	Number of drugs given during labor	continuous	
	Cesarean section	categorical	2
	Duration of first phase of delivery	continuous	
	Duration of second phase of delivery	continuous	
5 years	Housing conditions (Number occupants / room)	continuous	
	Months mother working during pregnancy	ordinal	3
	Parity	continuous	
	Social distress (composite score)	continuous	
	Domestic disputes	categorical	2

	Goodenough Draw-a-person test (DAP)	continuous	
	NDS coordination (composite score)	continuous	
	NDS behavior (composite score)	continuous	
	NDS total score	continuous	
	Dubowitz test total score	continuous	
	ITPA average score	continuous	
9 years	Social distress (composite score)	continuous	
	Special class	ordinal	4
	Teacher´s assessment of school achievement	ordinal	5
	Remedial tutoring	categorical	2
	Teacher's assessment of coordinative ability	ordinal	5
	Teachers assessment of restlessness	ordinal	5
	Teachers assessment of adjustment	ordinal	5
	Parents' assessment of school achievement	ordinal	5
	School grades	continuous	
	ITPA average score	continuous	
	WISC VIQ	continuous	
	WISC PIQ	continuous	
	WISC IQ	continuous	
	Parents' perceived benefit of participation in study	ordinal	3
	TOMI total score	continuous	
	Goodenough Draw-a-person test (DAP)	continuous	
16 years	Current school / occupational status	ordinal	4
	Occupational plan	ordinal	4
	Parents assessment	ordinal	5
	YSR sum score	continuous	
	YSR internalizing	continuous	
	YSR externalizing	continuous	

ITPA = Illinois Test of Psycholinquistic Ability, NDS = Neurodevelopmental Screening test, WAIS = Wechsler Intelligence Scale for Children, IQ = intelligence quotient, VIQ = visual intelligence quotient, PIQ = performance intelligence quotient, TOMI = Test of Motor Impairment, YSR = Youth Self Rating (Achenbach)
