# Supplementary material for: Attrition in a 30-year follow-up of a perinatal birth risk cohort: factors change with age
Source: PeerJ. 2014 Jul 8;2:e480. doi: 10.7717/peerj.480 (PMC4103077; doi:10.7717/peerj.480)
Supplement: Supplemental Information S8 — Variable importance given by random forest classification model, all variables included. [file peerj-02-480-s008.pdf]

Variable importance in random forest classification

	At birth		Age 5		Age 9		Age 16		Age 30	
Birth related	Birth Weight	1.00	Mother's age	1.00	Gestational weeks	0.52	Mother's age	0.56	Mother's age	0.87
	Mother's age	0.78	Birth Weight	0.69	Birth Weight	0.48	Birth Weight	0.37	Pregnancy complications	0.58
	Resuscitation	0.77	Hyperbilirubinemia	0.58	Mother's age	0.44	Pregnancy complications	0.31	Birth Weight	0.41
	Perinatal treatments	0.75	Pregnancy complications	0.56	Hyperbilirubinemia	0.40	Apgar 1 min	0.20	X-ray examination in pregnancy	0.26
	Gestational weeks	0.75	Gestational weeks	0.55	Respiratory problem	0.38	Apgar 5 min	0.16	Apgar 1 min	0.22
	Apgar 1 min	0.73	Perinatal treatments	0.48	Placental pathology	0.34	Apgar 15 min	0.14	Perinatal treatments	0.21
	Respiratory problem	0.63	Resuscitation	0.47	Neurological symptoms	0.34	X-ray examination in pregnancy	0.11	Apgar 15 min	0.19
	Hyperbilirubinemia	0.60	Apgar 1 min	0.45	X-ray examination in pregnancy	0.31	Perinatal treatments	0.11	Apgar 5 min	0.18
	Apgar 5 min	0.55	Respiratory problem	0.44	Cord pathology	0.30	Gender	0.08	Medications 1 and 2 trimester	0.18
	Apgar 15 min	0.44	Apgar 5 min	0.39	Apgar 1 min	0.29	Medications 1 and 2 trimester	0.07	Gender	0.17
	X-ray examination in pregnancy	0.44	X-ray examination in pregnancy	0.38	Perinatal treatments	0.28	Cesarean section	0.03	Cesarean section	0.10
	Delivery phase 2 (h)	0.42	Neurological symptoms	0.38	Toxemia	0.25				
	Neurological symptoms	0.37	Delivery phase 1	0.32	Amniotic fluid pathology	0.24				
	Delivery phase 1	0.37	Medications 1 and 2 trimester	0.31	Resuscitation	0.21				
	Toxemia	0.35	Placental pathology	0.30	Apgar 5 min	0.20				
	Placental pathology	0.33	Delivery phase 2 (h)	0.29	Apgar 15 min	0.16				
	Amniotic fluid pathology	0.32	Apgar 15 min	0.29	Medications in delivery	0.16				
	Miscarriages	0.28	Cord pathology	0.25	Miscarriages	0.14				
	Cord pathology	0.27	Toxemia	0.24	Cesarean section	0.09				
	Medications in delivery	0.27	Miscarriages	0.23	Gender	0.09				
	Gender	0.16	Amniotic fluid pathology	0.23						
			Medications in delivery	0.20						
			Cesarean section	0.14						
			Gender	0.13						

Variable importance in random forest classification

	At birth		Age 5		Age 9		Age 16		Age 30	
SES related	Distance to hospital	0.55	Distance to hospital	0.41	Housing conditions (5)	0.85	Childs' security (9)	0.44	Parents' assessment (16)	0.79
	Father's social class	0.54	Mother working in pregnancy	0.39	Parity (5)	0.80	Housing conditions (5)	0.33	Childs' security (9)	0.66
	Mother working in pregnancy	0.44	Father's social class	0.36	Domestic problem	0.53	Special education (9)	0.30	Special education (9)	0.37
	Smoking in pregnancy	0.35	Smoking in pregnancy	0.31	No of occupants	0.45	Father's social class	0.19	Current activity (16)	0.35
	Marital status	0.29	Marital status	0.23	Father's social class	0.45	Mother working in pregnancy	0.14	Subjective gain (9)	0.30
	Parity	0.17	Parity	0.13	Mother working (5)	0.45	Distance to hospital	0.11	Housing conditions (5)	0.27
			Domestic problem	0.08	Family's social situation (5)	0.38	Smoking in pregnancy	0.10	Occupational plan (16)	0.22
					Marital status	0.30	Parity	0.09	Father's social class	0.20
					Distance to hospital	0.25	Subjective gain (9)	0.07	Smoking in pregnancy	0.19
					Mother working in pregnancy	0.22	Mother working (5)	0.07	Mother working in pregnancy	0.17
					Smoking in pregnancy	0.22	Marital status	0.05	Distance to hospital	0.16
					Parity	0.10	Remedial tutoring (9)	0.04	Mother working (5)	0.14
					Domestic problem (5)	0.07	Father working (9)	0.04	Father working (9)	0.14
							Mother working (9)	0.03	Parity	0.09
									Mother working (9)	0.09
									Remedial tutoring (9)	0.06
									Marital status	0.06

Variable importance in random forest classification

	At birth	Age 5	Age 9		Age 16		Age 30	
Neurodevelopmental related			Dubowitz test (5)	1.00	NDS (5)	1.00	NDS (5)	1.00
			DAP (5)	0.90	DAP (9)	0.38	Dubowitz test (5)	0.54
			Coordination	0.72	WISC (9)	0.37	ITPA (5)	0.53
			ITPA (5)	0.72	TOMI (9)	0.33	*ITPA (9)*	0.48
			NDS (5)	0.71	*ITPA (9)*	0.32	WISC VIQ (9)	0.48
					WISC PIQ (9)	0.30	Drawing (5)	0.47
					ITPA (5)	0.30	WISC PIQ (9)	0.46
					Dubowitz test (5)	0.30	School grades (30)	0.45
					DAP (5)	0.29	Teacher's assessment (9)	0.44
					Drawing (5)	0.25	TOMI (9)	0.40
					WISC VIQ (9)	0.24	DAP (5)	0.40
					Teacher's assessment (9)	0.23	DAP (9)	0.40
							WISC (9)	0.39
Classification accuracy	0.81	0.74	0.90		0.76		0.96	
(95 % confidence limits)	0.78 - 0.82	0.71 - 0.76	0.87 - 0.91		0.72 - 0.79		0.94 - 0.97	

The most important variables at each age level selected for classification by the random forest classification model. Accuracy (overall fraction correct) calculated from 2 * 2 contingency table (a + d / t). Numbers in parenthesis refer to the age in which the variable is measured. BMI = body mass index, DAP = Draw A Person test, ITPA = Illinois test of psycholinguistic ability, TOMI = Test of motor impairment, NDS = Neurodevelopmental screen, WISC = Wechsler intelligence test for children, VIQ = verbal intelligence quotient, PIQ = performance intelligence quotient.
